# Automatic renal carcinoma biopsy guidance using forward-viewing endoscopic optical coherence tomography and deep learning

**DOI:** 10.21203/rs.3.rs-3592809/v1

**Published:** 2023-11-23

**Authors:** Qinggong Tang, Chen Wang, Haoyang Cui, Qinghao Zhang, Paul Calle, Yuyang Yan, Feng Yan, Kar-ming Fung, Sanjay Patel, Zhongxin Yu, Sean Duguay, William Vanlandingham, Chongle Pan

**Keywords:** Optical coherence tomography, Endoscope, Surgical Guidance, Renal cancer diagnosis, Deep learning

## Abstract

Percutaneous renal biopsy (PRB) is commonly used for kidney cancer diagnosis. However, current PRB remains challenging in sampling accuracy. This study introduces a forward-viewing optical coherence tomography (OCT) probe for differentiating tumor and normal tissues, aiming at precise PRB guidance. Five human kidneys and renal carcinoma samples were used to evaluate the performance of our probe. Based on their distinct OCT imaging features, tumor and normal renal tissues can be accurately distinguished. We examined the attenuation coefficient for tissue classification and achieved 98.19% tumor recognition accuracy, but underperformed for distinguishing normal tissues. We further developed convolutional neural networks (CNN) and evaluated two CNN architectures: ResNet50 and InceptionV3, yielding 99.51% and 99.48% accuracies for tumor recognition, and over 98.90% for normal tissues recognition. In conclusion, combining OCT and CNN significantly enhanced the PRB guidance, offering a promising guidance technology for improved kidney cancer diagnosis.

## 1 Introduction

Renal cancer is a disease characterized by the uncontrolled growth of kidney cells, resulting in the formation of tumor tissue. The lifetime risk of renal cancer was estimated to be ~ 2.02% in males and ~ 1.03% in females [1]. American Cancer Society projected approximately 81,800 new cases of renal cancer in the United States in 2023, including 52,360 males and 29,440 females, and 14,890 (9,920 males and 4,970 females) would die from it [1]. Renal cell carcinoma (RCC) is the most prevalent type of renal cancer, constituting approximately 95% of all renal cancer cases, and it has shown a consistent rise over the past few decades [2, 3]. Accurate identification and diagnosis of renal tumor malignancy is critical for devising effective treatment plans. At present, percutaneous renal biopsy (PRB) is the most commonly used procedure to extract renal tissue for pathological analysis [4]. Since being proposed in 1951, PRB has been widely applied in the diagnosis and prognosis of kidney diseases [5]. It has been considered as an effective method for the assessment of renal tumors from patients with RCC. During the PRB procedure, the patient is placed in a prone position. An exploratory needle is then inserted into the kidney to establish a path for the subsequent insertion of the biopsy needle [6]. The biopsy needle is employed to extract a tissue sample from the tumor. The obtained tissue sample is processed, analyzed, and classified as benign, malignant, or nondiagnostic for patients with RCC [7].

Despite being widely used for kidney disease diagnosis and renal tumor tissue evaluation, PRB still faces challenges in the tissue localization. It has been reported that even with the assistance of a radiologist, approximately 92% of PRBs required two or more attempts in order to accurately extract the targeted tissue [8]. More insertion attempts raise the likelihood of complications, including hematoma and infection [9, 10]. Therefore, accurate guidance of needle insertion is of great importance for effective and safe renal carcinoma biopsy. Conventional imaging modalities have been employed for PRB needle guidance. Ultrasound has been harnessed to assist in needle guidance, leading to a significant reduction in the incidence of severe PRB complications over the past few decades [11, 12]. Computed tomography (CT) has also been utilized in the diagnosis of metastatic lesion during percutaneous biopsy [13]. Three-dimensional cone-beam CT guidance has been documented as beneficial for the secure and effective determination of needle trajectory, as well as for performing biopsies on small renal masses [14]. Moreover, the guidance of PRB through magnetic resonance imaging (MRI) exhibits promising outcomes in precisely targeting the renal tumor and accurately locating the PRB needle tip, particularly in obese patients [15]. Although these macroscopic imaging methods, including ultrasound, CT and MRI, are effective in needle trajectory determination, they cannot accurately identify tissue types ahead of the needle tip due to their limited spatial resolutions [16]. As a result, it was reported that ~ 10% – 20% of the biopsy results were non-diagnostic due to the limitation of imaging guidance of tumor location [16]. Hence, there is an unmet need for an imaging method capable of accurately identifying tissue types ahead of the PRB needle.

Optical coherence tomography (OCT) is a well-established imaging technique with micron-level spatial resolution [17]. Therefore, OCT has the potential for providing enhanced fine-scale visualization during PRB needle guidance. Moreover, OCT has already demonstrated feasibility in diagnosing renal masses, with the ability to differentiate renal tumor tissue from normal renal tissue based on distinct attenuation coefficients in OCT images [18]. OCT can also provide real-time imaging [19]. Therefore, we hypothesized that OCT can be used in the real-time guidance of the PRB needle by detecting and identifying the renal tumor tissue. To enable tissue imaging ahead of the PRB needle, we have developed a forward-view OCT probe that incorporates gradient-index (GRIN) lenses functioning as a forward-viewing endoscope. Our previous work successfully demonstrated the application of endoscopic OCT in percutaneous nephrostomy (PCN) guidance, accurately identifying various kidney tissues within pig models [20, 21]. Hence, the OCT probe system holds the promise in PRB needle guidance and renal tumor recognition.

Because visually identifying tissue from OCT images incurs a significant learning curve for radiologists and PRB operators, we have developed automatic imaging processing techniques, including tissue classification using attenuation coefficient and deep learning methods. Mapping optical attenuation coefficients has already been employed in distinguishing between cancerous and non-cancerous tissues [22]. Given that cancerous tissue tends to exhibit higher density in the imaging results, its attenuation coefficient typically surpasses that of normal tissues. Therefore, attenuation coefficient can be a promising feature used for tumor tissue recognition during PRB needle guidance. Furthermore, deep learning has been demonstrated to be an effective approach in tissue classification and recognition for surgical guidance [21, 23, 24]. In this study, we have applied convolutional neural networks (CNNs) for automatic identification of normal renal tissue and kidney tumor. The performance of these methods was benchmarked for renal tissues classification and tumor diagnosis.

## 2 Materials and Methods

### Experimental setup

**Figure 1:**
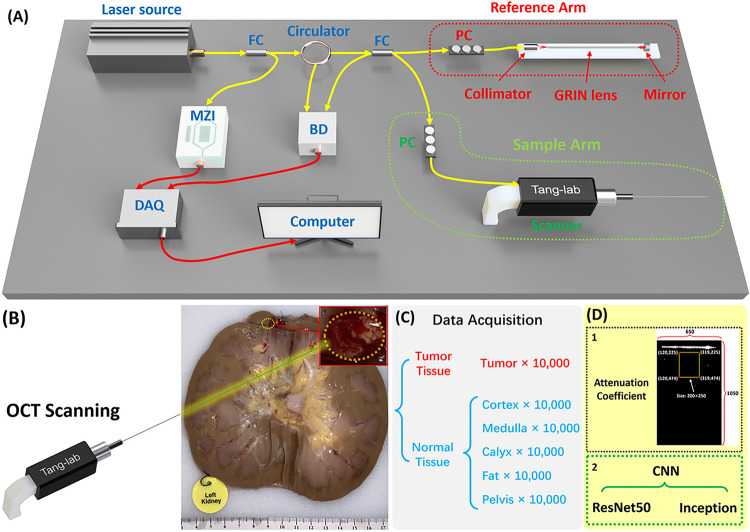
(A) Schematic of the forward-viewing OCT probe. FC: fiber coupler;PC: polarization controller; MZI: Mach–Zehnder interferometer; BD: balanced detector; DAQ: Data acquisition. (B) Picture of the human kidney with tumor. (C) Data acquisition procedure. (D) Two classification methods used for tissue classification.

We constructed our forward-viewing optical coherence tomography (OCT) probe based on a swept-source OCT (SS-OCT) system [21, 24, 25]. The light source utilized was a 1,300 nm swept-source laser with a bandwidth of 100 nm. The scanning rate of our system reached 200 kHz. As illustrated in Fig. 1, the light was initially divided using a fiber coupler (FC) into two distinct paths: one directed with 97% of power towards a circulator for the OCT imaging, and the other with 3% power directed to a Mach-Zehnder interferometer (MZI) to initiate the sampling process. Upon traversing the circulator, the laser underwent another division through another FC into the reference arm and sample arm. Subsequently, the light reflected by the mirror in the reference arm and the light backscattered by the tissue in the sample arm converged, generating an interference signal. This signal was then denoised using a balanced detector (BD), then acquired by the data acquisition (DAQ) board and processed and visualized on a computer in the end. Polarization controllers (PC) were employed in both arms to mitigate background noise.

In PRB procedures, 14 gauge (G) or 16 G biopsy needles are commonly used [26]. To assess feasibility, we employed GRIN lenses with a diameter of 1.3 mm and length of 138.0 mm, allowing them to be accommodated within the 14 G or 16 G biopsy needles. These GRIN lenses possess a refractive index distribution that varies perpendicular to the optical axis, facilitating effective image transmission from one end to the other [27].

In our configuration, we positioned a GRIN lens in the sample arm ahead of the OCT scanning lens for endoscopic imaging. Additionally, another same GRIN lens was situated in the reference arm to counteract dispersion. The proximal end of the GRIN lens in the sample arm was positioned at the focal point of the scanner lens, maximizing the delivery of light to the sample. Our system features an axial resolution of approximately 11 μm and a lateral resolution of around 20 μm. It provides a sensitivity of 92 dB and covers a field of view (FOV) of approximately 1.25 mm in diameter, rendering it well-suited for our intended application.

### Data acquisition

The study was approved by the University of Oklahoma Institutional Review Board (Study number: 12462). Written informed consent was obtained by Lifeshare of Oklahoma. Patient demographics were collected upon obtaining consent. For our dataset, we utilized five human kidney samples and five renal carcinoma samples. OCT images of carcinoma tissue and different normal tissues were imaged by our OCT probe. To keep the organ as fresh as possible, the kidney was preserved by hypothermic machine perfusion (HMP), and we started the experiment as soon as the sample was acquired.

Figure 1(B) illustrates one kidney sample with carcinoma, clearly showcasing the tumor tissue alongside other normal renal tissues including cortex, medulla, calyx, fat, and pelvis. To emulate a practical biopsy procedure during the experiment, we carefully inserted the OCT probe into different renal tissue types, applying controlled force to compress the tissue. OCT image acquisition was successfully performed in the final.

For quantitative identification of tumor tissue, we utilized both conventional methods, involving the calculation of attenuation coefficients, and deep learning techniques for automated recognition of renal tissue types. To train the machine learning model, we obtained a dataset of 10,000 OCT images from the tumor and five different normal renal tissues, respectively (Fig. 1(C)). The size of all output images was set as lateral X depth: 650×1050 (X×Z: 1.30 mm×2.10 mm), so each result covered the same FOV for attenuation coefficient method and CNN process (Fig. 1(D)).

### Attenuation coefficient method for tissue recognition

Our OCT findings provided in-depth structural insights into renal tissues. The attenuation coefficient serves as a crucial parameter, signifying the decrease in signal intensity observed in OCT images as depth increases [28]. In structural OCT images, the imaging intensity reflects the backscattering level of the tissue sample, so different attenuation coefficients demonstrate different tissue features and can be used for tissue classification. The use of attenuation coefficient for cancer detection has been extensively documented [29–31]. Compared to normal tissues, cancerous tissue is typically denser thus its imaging signal value attenuated faster in vertical direction and represented by higher attenuation coefficient. The attenuation coefficient of each A-scan can be extracted and used to generate a spatial attenuation coefficient distribution, which helps visually distinguish tumor and normal tissues [22].

Here we used Beer-Lambert law to describe the structural OCT intensity signals in depth as follows:

1 $$I_{\left(z\right)}=I_{0}e^{-2\mu z}$$

where $I_{0}$ represents the initial incident light intensity, μ is the attenuation coefficient value which represents the decay rate, and z is the depth [32]. Thus, μ can be described as:

2 $$\mu =-\frac{1}{2}\cdot \frac{d\log I_{\left(Z/0\right)}}{d_{z}}$$

$I_{\left(Z/0\right)}$ is the ratio of recorded OCT intensity in depth to the initial incident light intensity. The intrinsic optical attenuation coefficient was calculated according to the slope of OCT intensity in a 250-pixel depth window and then averaged in a 200-pixel width region as shown in Fig. 1(D).

### CNN methods for tissue identification

Two CNN architectures, ResNet50 and InceptionV3, were evaluated. ResNet50 has ~ 25.6 million parameters and InceptionV3 has ~ 22 million parameters. We used a nested 4-fold cross-validation and 5-fold cross-testing procedure [21], in which each iteration rotated three subjects to the training dataset, one subject to the validation dataset, and one subject to the testing dataset. This division enabled us to obtain a more accurate estimation of the generalization error, as every subject was included in the testing dataset once. Early stopping was implemented with a patience of 20. The optimizer used for the training was stochastic gradient descent with Nesterov accelerated gradient, utilizing a momentum value of 0.9 and a learning rate of 0.01. Additionally, the learning decay rate was set to be 0.1. The average epoch count from the cross-validation folds was subsequently employed to train the model intended for use on the test dataset. The test performances of the obtained models were benchmarked using the 5-fold cross-testing. The classification performance was measured using four metrics, including accuracy, precision, recall, and F_1_ score as defined below:

3 Accuracy=(TP+TN)/(TP+FP+TN+FN)

4 Precision=TP/(TP+FN)

5 Recall=TP/(TP+FN)

6 $$F_{1}\text{score}\;=2•\frac{\text{Precision}•\mathrm{Recall}}{\mathrm{Precision}+\mathrm{Recall}}=\frac{TP}{TP+\frac{1}{2}(FP+FN)}$$

where TP is true positive, FP is false positive, TN is true negative, and FN is false negative.

To interpret the prediction of deep learning models [33, 34], GradCAM [35] was used to generate pixel saliency heatmaps. One of the five subjects’ images were used to generate the heatmaps for both CNN models: InceptionV3 and ResNet50. For example, all images of the cortex from the chosen subject were utilized. We calculated the sum of the corresponding pixel points of the images, then the sum was divided by the number of total images used, resulting in an average heatmap image of cortex. Same practice applied to the rest of the tissue types.

## 3 Results

### 3.1 Imaging results of renal tumor and normal tissues

**Figure 2:**
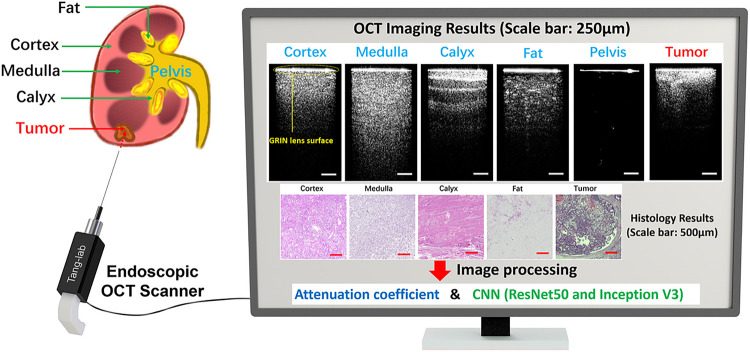
OCT imaging results of five normal tissues and tumor tissue (Scale bar: 250μm).Corresponding histology results (Scale bar: 500μm).

Figure 2 illustrates the OCT imaging results obtained through our OCT probe, showcasing both renal carcinoma tumor and five distinct normal renal tissues. Two dimensional (2D) cross-sectional OCT images of these tissues demonstrate different structural features. The corresponding histology results are also shown in Fig. 2.

Figure 2. OCT imaging results of five normal tissues and tumor tissue (Scale bar: 250μm). Corresponding histology results (Scale bar: 500μm).

In the OCT results, there is a bright line on the top (pointed by a yellow arrow) which corresponds to the surface of the GRIN lens. The intensity signal distributions of both cortex and medulla are relatively homogeneous, although the imaging depth of medulla surpasses that of cortex. Calyx exhibits alternately shaded stripes, a result of its transitional epithelium and fibrous tissue composition. Fat presents the darkest pattern, accompanied by bright dots characteristic of adipocytes. We injected synthetic urine into the pelvis and tied the ureter during the experiment and inserted the probe into the pelvis for imaging. As an empty space for collecting urine, the pelvis has no tissue signal under the GRIN lens surface. The renal tumor, being the pivotal tissue type for identification during PRB, showcases the shallowest imaging depth while presenting the highest brightness among all OCT results. Its structural features exhibit irregularities. The corresponding histology results were also presented in Fig. 2. These tissues showed different tissue structures and distributions and correlated well with their OCT results. Different normal renal tissues and tumor tissues can be categorized based on their distinctive OCT imaging features. As a result, our endoscopic OCT probe has the potential to assist in the recognition of tissue types ahead of the needle during PRB procedures.

### 3.2 Classification using attenuation coefficient method

We first assessed the performance of attenuation coefficient method in distinguishing renal tumor and normal tissues. The attenuation coefficient did not work for the pelvis, because the values of most pixels were zero and the logarithm could not be calculated. Therefore, only tumor tissue and other four normal tissue types were tested using the attenuation coefficient method.

This study involved the processing of a total of 50,000 OCT images (after removing pelvis category), with each tissue type contributing 10,000 images. The performance of the results was benchmarked using the cross-testing procedure. Four kidneys were randomly selected as the training dataset, while the remaining kidney served as the testing dataset. To calculate the attenuation coefficient, the region of interest (ROI) window was selected to be 200×250 pixels for each OCT image. The ROIs were chosen from the central sections of the images and the coordinate of the area is selected in the top middle position which includes most tissue information (Fig. 1(D)). The mean value of all the attenuation coefficient results from every vertical direction was used as the attenuation coefficient level for each OCT image.

**Figure 3:**
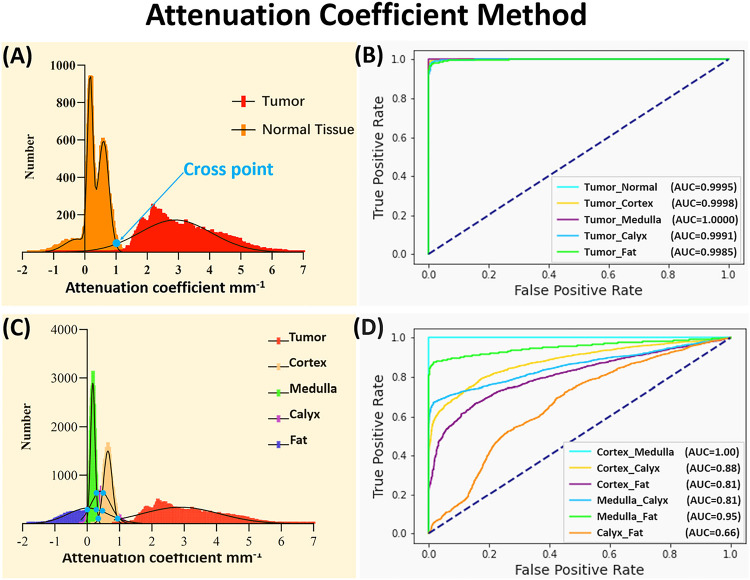
(A) Attenuation coefficient distribution results between normal tissue and tumor tissue. (B) ROC curves of the classification with attenuation coefficient between tumor tissue and other tissues. (C) Attenuation coefficient distribution results of all five tissue types. (D) ROC curves of the classification with attenuation coefficient among different normal tissues.

Fig. 3 displays the attenuation coefficient distributions for the five tissue types (four normal renal tissues and tumor tissue). Firstly, considering cortex, medulla, calyx, and fat are all normal tissues, these four types were initially consolidated into a single classification. In this testing, all the tumor tissue images from 5 samples (50,000 images in total) were utilized. To make the number of normal tissue images equal to the number of tumor images, we randomly selected 2,500 images from each normal tissue in every sample (2,500 images × 4 tissue types × 5 samples = 50,000 images). In general, the tumor tissue and the normal tissues displayed distinctive distribution characteristics, thereby enabling a clear differentiation between tumor and normal tissues as shown in Fig. 3(A). Normal distributions were fitted to the attenuation coefficient distributions as shown in Fig. 3(A). To quantify the separation of the tumor from normal tissues, the cross point (blue dot) of two curves was chosen as threshold and used to evaluate the accuracy. The receiver operating characteristic (ROC) curves were shown between tumor and normal renal tissues in Fig. 3(B). The area under the ROC curves (AUC) (Tumor_Normal) was 99.95%, which validated the tumor recognition accuracy based on attenuation coefficients.

We then evaluated classification of the renal tissue types by using 10,000 OCT images from 5 samples (50,000 images in total). The attenuation coefficient distributions were shown in Fig. 3(C). To quantify the separation of the tumor from each normal tissue (i.e., tumor vs cortex, tumor vs medulla, tumor vs calyx, and tumor vs fat), the cross point (blue dot) of two curves was again chosen as threshold and used to evaluate the accuracy. The AUCs for differentiating the tumor tissue from each normal renal tissue were all over 99.85% (Fig. 3(B)), which further proved the tumor recognition capacity of using attenuation coefficients.

However, different normal renal tissues were difficult to classify using attenuation coefficient method. The AUCs were 100% for cortex vs. medulla, 88% for cortex vs. calyx, 81% for cortex vs. fat, 86% for medulla vs. calyx, 95% for medulla vs. fat, and only 66% for calyx vs. fat (Fig. 3(D)). The confusion matrix for multi-class classification was further shown in Table 1(A), and the corresponding prediction results were shown in Table 1(B). Tumor demonstrated high recognition performance, with 98.19% accuracy, 91.85% precision, 99.83% recall, and 95.67% F_1_ score. However, the normal tissue recognition was challenging, especially for calyx (78.77% accuracy, 43.18% precision, 19.47% recall, and 26.84% F_1_ score). The needle’s precise placement can enhance surgical efficiency and overall effectiveness. The present results primarily contributed to tumor recognition, but the performance in identifying other normal kidney tissues still needed improvement. In particular, the attenuation coefficient methods misclassified over 500 images of calyx as tumor tissue, causing potential sampling errors.

### 3.3 Tissue recognition results using CNN

**Figure 4:**
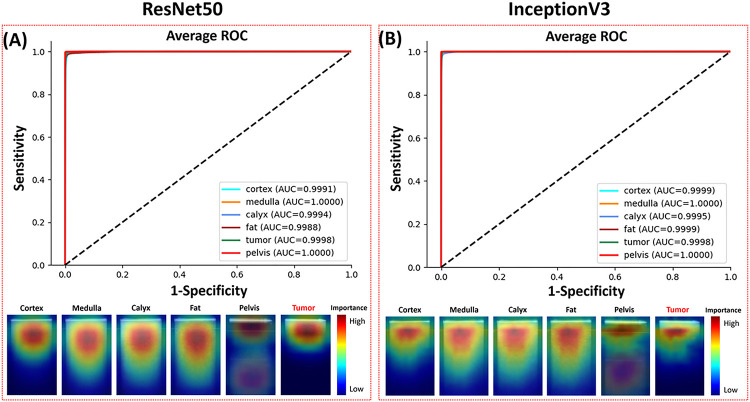
ROC curves and pixel importance heatmaps of (A) ResNet50 and (B) InceptionV3.

The recognition of kidney tissues using the ResNet50 and InceptionV3 CNNs was evaluated using nested cross-validation and cross-testing. The ROC curves and pixel saliency heatmaps were shown in Fig. 4. The confusion matrixes and performance metrics were shown in Table 2.

The ResNet50 CNN yielded a tumor recognition accuracy of 99.51%. For normal tissue recognition, the prediction accuracies were 98.96% for cortex, 99.78% for medulla, 99.09% for calyx, 99.27% for fat, and 99.95% for pelvis. 97.72% of the misclassifications of tumor samples were erroneously categorized as cortex. None of cortex, medulla and fat images were predicted to be tumor, and 1.48% of the calyx images was falsely predicted as tumor. The InceptionV3 CNN reached 99.48% tumor prediction accuracy. The accuracies for normal tissue classification were 99.58% for cortex, 99.88% for medulla, 99.27% for calyx, 99.65% for fat, and 99.95% for pelvis. Calyx recognition has the lowest accuracy, and 0.02% of tumor samples were erroneously categorized as calyx, which is different from the ResNet50 model. Both ResNet50 and InceptionV3 achieved >99.9% ROC AUC for all tissues.

InceptionV3 outperformed ResNet50 in overall precision for the classification of the normal renal tissues, while with a slightly lower precision in the tumor classification. All normal tissues and tumor results using InceptionV3 have higher recalls than ResNet50 except for calyx.

All computations were conducted on a server equipped with two NVIDIA RTX A6000 graphics cards. The cross-validation required an average of 1,435 minutes per fold. The average inference time over 320 images was 18 milliseconds per image for ResNet50 and 11 milliseconds per image for InceptionV3.

The average pixel importance heatmaps of all the images from the two models were shown in Fig. 4. These heatmaps highlighted the regions important for the models to distinguish different tissue types. The narrow area below the GRIN lens surface line was important for the tumor recognition. The important area for the pelvis recognition included a narrow part on the top and a wide area on the bottom. Heatmaps of the other four tissues all highlighted the top half of the images with strong signals. The important areas used by ResNet50 were sparser and wider than those used by InceptionV3. This could be attributed to the fact that ResNet50’s feature map was more focused and localized within each residual block than InceptionV3, so it could capture fine-grained details in the images in pixel level.

## 4 Discussion

Although PRB is the most commonly performed procedure for evaluating renal tissue, it remains a challenge even for experienced urologists to precisely extract tumor tissue with minimal sampling error. In this study, we tested the feasibility of using a forward-viewing OCT probe for RCC detection on *ex-vivo* human kidney for the first time. Five human kidneys with renal carcinoma were utilized in our experiments to validate our approach. The renal tumor tissue and different normal renal tissues, including cortex, medulla, calyx, renal fat, and pelvis were all included to evaluate the recognition accuracy of our system. We applied the probe on these tissues and obtained their OCT images. The OCT images of different renal tissues could be classified based on their distinct imaging characteristics. Moreover, the OCT probe’s dimensions were designed to fit inside the PRB needle’s hollow bore, enabling seamless integration without introducing any additional invasiveness. Under clinical settings, our OCT probe can image the tissue in front of the PRB needle in real time, effectively identifying the tissue type at the needle’s tip.

To automate the process of tissue identification, we utilized and compared two classification methods in our study: attenuation coefficient and deep learning. The attenuation coefficient method has been widely used in OCT-based cancer diagnosis, including breast cancer [30], prostate cancer [36], bladder cancer [31], colon cancer [37], etc. Renal mass diagnosis using OCT was also reported, and it exhibited highly promising outcomes in predicting RCC [38]. For the classification task, we imaged 10,000 OCT images from renal tumor, cortex, medulla, calyx, renal fat, and pelvis, respectively. Our study evaluated the attenuation coefficient for identification of tumor and normal tissues. Discrimination of tumor against other normal tissues was promising with the accuracy of 98.19%. While the results were generally favorable, misrecognition still happened. Additionally, relying solely on the attenuation coefficient proved challenging when it came to distinguishing between different types of normal renal tissues. The classification accuracy results were not satisfactory, particularly for the calyx prediction accuracy which fell below 80%.

Accurate identification of normal renal tissues prior to biopsy needle insertion played a pivotal role in precisely tracking the needle’s location and avoiding sampling error. To address this critical need, we further evaluated the deep learning methods in our study. Specifically, we employed two well-established CNN architectures, ResNet50 and InceptionV3, both of which demonstrated robust performance in tumor recognition and the classification of various normal renal tissues.

Remarkably, ResNet50 and InceptionV3 achieved tumor recognition rates of 99.51% and 99.48%, respectively, both of which surpassed the performance of the attenuation coefficient method. Equally impressive was the accuracy in normal tissue recognition, with rates consistently exceeding 99% across all tissue types in both models (except cortex prediction using ResNet50 of 98.96%). ROC curves for both models showed AUC values consistently exceeding 99.9%. This observation underscores the effectiveness of the CNN-based approach for the classification of different normal renal tissues compared to the attenuation coefficient method. The outstanding performance of these models can be attributed, in part, to their substantial parameter sets. ResNet50 and InceptionV3 feature 25.6 million and 22 million parameters, respectively, in contrast to the single parameter employed by the attenuation coefficient method. To further expedite processing times, future experiments could be conducted for the use of smaller architectures without compromising performance.

## 5 Conclusion

We tested the feasibility of employing an endoscopic OCT probe for the diagnosis of renal cancer. Renal tumor and normal kidney tissues can be distinguished based on their OCT images. To automate this process, we utilized both attenuation coefficient and deep learning methods for renal tissue recognition. The attenuation coefficient enabled the differentiation between tumor and normal tissues, while the CNN method further classified tumor tissue as well as different normal renal tissues accurately. By combining the deep learning approach with the forward-viewing OCT probe, we envision the creation of a precise imaging guidance tool for the PRB procedure. Moreover, our system can complement the established clinical PRB guiding methods, including ultrasound, fluoroscopy, and CT by providing high-resolution images in front of the PRB needle in real time.

## Figures and Tables

**Table 1.** (A) Confusion matrix of the averaged five kidneys. (B) Prediction results: Accuracy; Precision; Recall; and F1 Score of different tissue types.

**(A)**	Tissue		Prediction				
			Cortex	Medulla	Calyx	Fat	Tumor
	True	Cortex	**9145**	14	723	0	118
		Medulla	3	**9154**	756	87	0
		Calyx	4326	2820	**1947**	347	560
		Fat	1895	1872	1083	**4942**	208
		**Tumor**	17	0	0	0	**9983**

(B)	Tissue	Accuracy	Precision	Recall	F_1_ Score
	Cortex	85.81%	59.44%	91.45%	72.05%
	Medulla	88.90%	66.05%	91.54%	76.73%
	Calyx	78.77%	43.18%	19.47%	26.84%
	Fat	89.02%	91.93%	49.42%	64.28%
	**Tumor**	**98.19%**	**91.85%**	**99.83%**	**95.67%**

**Table 2.** Confusion matrixes and performance metrics of (A) ResNet50 and (B) InceptionV3.

(A)	Tissue		Prediction of ResNet50						(B)	Tissue		Prediction of Inception V3					
			Cortex	Medulla	Calyx	Fat	Pelvis	Tumor				Cortex	Medulla	Calyx	Fat	Pelvis	Tumor
	True	Cortex	9875	75	23	27	0	0		True	Cortex	9964	1	1	34	0	0
		Medulla	43	9956	1	0	0	0			Medulla	15	9978	7	0	0	0
		Calyx	136	15	9553	148	0	148			Calyx	69	38	9639	0	0	254
		Fat	199	0	56	9745	0	0			Fat	121	0	30	9822	0	27
		Pelvis	0	0	21	0	9969	10			Pelvis	0	11	15	1	9973	0
		**Tumor**	**123**	**0**	**0**	**9**	**2**	**9866**			**Tumor**	**10**	**0**	**22**	**0**	**0**	**9968**

	Tissue	Accuracy	Precision	Recall	F_1_ Score		Tissue	Accuracy	Precision	Recall	F_1_ Score
	Cortex	98.96	95.19	98.75	96.92		Cortex	99.58	97.89	99.64	98.76
	Medulla	99.78	99.10	99.56	99.33		Medulla	99.88	94.50	99.78	99.64
	Galyx	99.09	96.95	95.53	97.21		Calyx	99.27	99.22	94.39	96.74
	Fat	99.27	98.15	97.45	97.80		Fat	99.65	99.64	98.22	98.92
	Pelvis	99.95	99.98	99.69	99.83		Pelvis	99.95	100.00	99.73	99.86
	**Tumor**	**99.51**	**98.43**	**98.66**	**98.54**		**Tumor**	**99.48**	**97.26**	**99.68**	**98.46**
